# A Simplified Understanding of the Black Swan: Anti-phospholipid Antibody Syndrome

**DOI:** 10.31729/jnma.4226

**Published:** 2019-04-30

**Authors:** Binit Vaidya, Shweta Nakarmi, Rakshya Joshi, Rikesh Baral

**Affiliations:** 1 Department of Rheumatology, National Center for Rheumatic Diseases, Ratopul, Kathmandu, Nepal

**Keywords:** *anticoagulants*, *anti-phospholipid syndrome*, *obstetric APS*, *thrombotic APS*

## Abstract

Anti-phospholipid Antibody Syndrome or Hugh's syndrome is a heterogeneous disorder, first fully described in 1980s. The syndrome is caused by the presence of specific antibodies against phospholipid binding plasma proteins in the serum of the patient, with or without underlying autoimmune diseases, that causes prolongation of tests of coagulation. High index of clinical suspicion is required for the diagnosis of Anti-phospholipid Antibody Syndrome. Stroke or myocardial infarction in young, unprovoked recurrent deep vein thrombosis and recurrent pregnancy loss are typical scenarios where Anti-phospholipid Antibody Syndrome should be suspected. Presence of non-criteria manifestations like livedo reticularis, skin ulcers, nephropathy, valvular heart disease and thrombocytopenia adds to the diagnostic clue for the presence of Anti-phospholipid Antibody Syndrome. Treatment of Anti-phospholipid Antibody Syndrome has preventive and therapeutic aspects that usually focus on thrombotic and obstetric manifestations of the disease. Therapeutic anti-coagulation with heparin followed by warfarin is required for patients presenting with acute thrombosis. Those with venous thrombosis are given moderate intensity warfarin (International Normalized Ratio, 2–3), whereas those with arterial thrombosis or recurrent venous thrombosis even on warfarin are treated with high intensity warfarin (International Normalized Ratio, 3–4). Similarly, anticoagulation with heparin is advised in patients with obstetric Anti-phospholipid Antibody Syndrome throughout pregnancy and up to six weeks postpartum. Treatment recommendations are still not clear for asymptomatic Anti-phospholipid Antibody Syndrome positive patients and in those with non-criteria manifestations of the disease. Steroids, intravenous immunoglobulin and immunosuppressant are reported to be effective in severe cases of catastrophic antiphospholipid syndrome characterized by rapid small vessel thrombotic involvement of multiple organ systems. Studies are evaluating the efficacy of direct thrombin inhibitors in the management of refractory cases.

## INTRODUCTION

Anti-phospholipid syndrome (APS) or Hugh's syndrome is an acquired thrombophilic condition of autoimmune origin characterized by vascular thrombosis, pregnancy morbidity and persistent positivity for antiphospholipid antibodies (aPL) with or without various non-criteria manifestations of the disease.^1^ It might be isolated primary disease or can be associated with underlying autoimmune diseases, mainly lupus. The condition is usually diagnosed in cases of unexplained thrombotic events or pregnancy losses with high index of clinical suspicion. The presence of serological evidence of antibodies against various phospholipids and phospholipid binding plasma proteins are essential for the diagnosis of APS.^1^ Description of the condition has been associated with many paradox and misnomers. The increased risk of thrombosis in the presence of coagulation inhibitor and thrombocytopenia is intriguing. The identification of various pathogenic antibodies adds to diagnostic confusion. Moreover, the treatment guidelines focus mainly on prevention and treatment of thrombotic manifestations of the syndrome and on reducing pregnancy morbidity.^2^ This review consolidates the current understanding of this Black Swan disease.

## THE HISTORY

The discovery of APS dates back to 1950s with the detection of prolonged tests of coagulation as activated partial thromboplastin time (aPTT) in patients with lupus.^3^ The immunological mechanism was suspected when most of these patients who had biological false positive serological test for syphilis (BFP-STS), which was used for population screening at that time, were often associated with other infectious diseases.^4^ When followed for certain period of time, some of these individuals were at higher risk for developing lupus.^5^ Some authors demonstrated that these patients had circulating antibodies which predisposed them to thromboembolic events.^6^ Also a few authors reported recurrent miscarriages in women with BFP-STS.^7^ The name lupus anticoagulant (LAC) was given to this coagulation inhibitor as it was detected in lupus patients.^8^ However, later the presence of such coagulation inhibitor was noted even in patients without lupus.^9^

A major milestone in the diagnosis of APS occurred when cardiolipin was identified as an antigen for the coagulation inhibitor and tests for detection of anti-cardiolipinantibodies (aCLs) was reported.^10,11^ Though initially derived from beef heart extract, cardiolipin was later found to be a phospholipid on inner mitochondrial membrane. Presence of antibodies to this membrane phospholipid could be detected by solid phase assay. With the ease and increasing availability of the test, issues were raised regarding the validity and reproducibility of the tests performed at different centers. Also, it was seen that these aCLs were detected in other conditions as well (eg. syphilis, leprosy, malignancies etc.).^12^ Much work was then carried out to increase the specificity of these antibodies. The results of this effort lead first to the use of titers of aCL and second to the detection of β2-glycoprotein I (β2 GPI) antigen.^13^

The (β2 GPI) is a plasma protein that binds to membrane phospholipids. It was subsequently realized that most of the LAC and aCL activity was actually directed against this antigen.^13^ However, few patients positive for anti-β2 GPI antibodies were negative for LAC and vice-versa. Many other phospholipids and phospholipid-protein complexes like prothrombin, phosphatidylserine, phosphatidylethanolamine etc. have been identified as potential antigen for aPL.^14^ The tests for these antibodies, however, have not been standardized for routine clinical use. Recent studies have shown that anti-domain I antibodies to β2 GPI are even more specific for detecting thrombotic APS.^15–17^ We can presume that as the understanding of the pathophysiology becomes elucidated, more and more specific tests and targeted treatments will be developed.

## PATHOPHYSIOLOGY

The “two hit” model of thrombosis is the most accepted theory where the first hit creates a thrombotic milieu by damaging the endothelium and the second hit triggers the coagulation process.^18,19^ β2-glycoprotein I is not only implicated as aPL binding protein but also has an orchestrating role in the thrombotic process of APS. This protein has five domains (I to V) with domain I binding to domain V and forming a circular form in the circulation. The aPL binding antigenic site, located in domain I, is hidden in this circular form. After an oxidative injury or stress, the disulfide bonds in the domains are broken and the protein opens up to a fishhook appearance exposing the cryptic aPL binding sites in domain I.^20^ This conformation of β2 GPI binds only to the stimulated endothelium (first hit).

This stimulation is due to infection or trauma in catastrophic APS; however, no clear mechanism is known for thrombotic APS. More plausible postulated theories include reduced activity of endothelial cell nitric oxide and oxidative injury to the endothelial cells.^21^

Binding of β2 GPI dimers to the phospholipids of endothelial cells and then the binding of aPL to domain I of β2 GPI orchestrates a mechanism which leads to activation of complement, activation of monocyte to produce more tissue factors and activation of platelets via mitogen-activated protein kinase pathways to increase thromboxane formation: all resulting in a prothrombotic mileu.^18,22^

The pathophysiology of obstetric APS is much less understood. Initial theories postulated the thrombotic occlusion of spiral arteries of placenta as plausible mechanism.^23^ However, it was realized that not all products of conception showed evidence of thrombosis or infarct. Also, many miscarriages were reported before the time of implantation. It is now understood that placental injury is mediated by two main mechanisms in addition to thrombosis. Firstly, complement activation leading to direct complement mediated injury of placenta and fetus^24^ and secondly, by direct inhibition of phospholipids on cytotrophoblast membrane, thus preventing the formation of syncytotrophoblast and finally leading to placental failure. It is also thought that reduction of annexin V on placental villi of women with antiphospholipid antibodies causes recurrent spontaneous abortion^25^ and antiphospholipid antibodies limit trophoblast migration by reducing IL-6 production and stat3 activity.^26^

**Figure 1.**
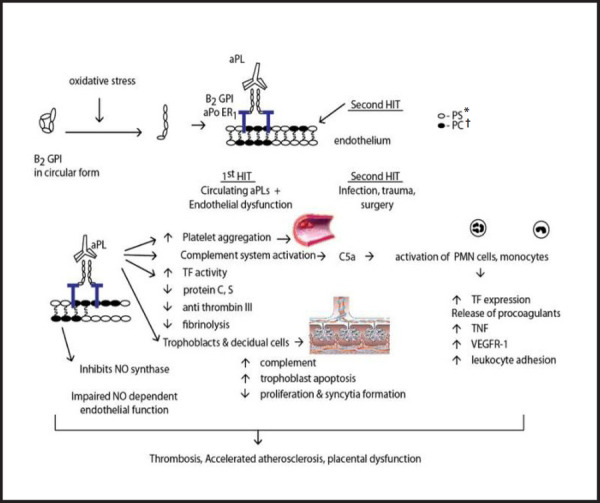
Pathophysiology of antiphospholipid induced thrombotic and obstetric manifestations.

The pathophysiology of thrombotic and obstetric manifestations initiated by aPLs are summarized (Figure 1).

**Table 1:** Revised Sapporo classification criteria or the Sydney criteria.^1^

Clinical criteria	
Vascular thrombosis	Pregnancy morbidity
• One or more clinical episodes of venous, arterial or small vessel thrombosis, with the exception of superficial venous thrombosis in any tissue or organ. Thrombosis must be confirmed using Doppler or imaging studies or histopathology. For histopathologic confirmation, thrombosis should be present without significant evidence of inflammation in the vessel wall.	One or more unexplained deaths of a morphologically normal fetus at or beyond 10^th^ week of gestation. One or more premature births (<34 weeks of gestation) of a morphologically normal neonate, because of eclampsia, severe pre-eclampsia and placental insufficiency. Three or more unexplained consecutive spontaneous abortions before the 10^th^ week of gestation (excluded maternal anatomic or hormonal abnormalities and chromosomal cause).
**Laboratory criteria**	
Lupus anticoagulants (LA) detected according to the guidelines of the International Society on Thrombosis and Haemostasis (Scientific Subcommittee on Las/phospholipid-dependent antibodies) is considered positive if present in plasma, on two or more occasions at least 12 weeks apart. Anticardiolipin (aCL) antibodies of IgG and/or IgMisotype measured by a standardized ELISA are considered positive if present in serum or plasma, in medium or high titre (i.e., >40 GPL or MPL, or 99^th^ centile), on two or more occasions, at least 12 weeks apart. Anti-β_2_ glycoprotein-I antibodies (aβ_2_GPI) of IgG and/or IgMisotype measured by a standardized ELISA, according to recommended procedures, are considered positive if present in serum or plasma, in titre >99^th^ centile, on two or more occasions, at least 12 weeks apart.	

β2GPI normally occurs in circular form in the circulation. The cryptic antigenic focus located on domain I is exposed when oxidative stress opens the circular form by breaking the disulfide bonds. The aPL then binds to the β2GP1 and the complex binds to stimulated endothelium orchestrating the mechanism of inflammation and thrombosis.

## DIAGNOSIS

The diagnosis of APS requires the presence of at least one clinical event of thrombosis or pregnancy loss and positivity for at least one of the tests for aPL on two separate occasions. The two separate occasions were defined as at least 8 weeks in the original Sapporo criteria founded in 1996 at Sapporo, Japan.^27^ However, in 2006 modification of criteria made in Sydney, the duration was increased to 12weeks to increase the specificity of the diagnosis. Also, in addition to venous and arterial thrombosis, histologically proven small vessel thrombosis was also included in the criteria.^1^ The Sydney criteria have been endorsed in the 14^th^ international congress on APS (Table 1).^28^

Other changes that were made in revised criteria included inclusion of anti-β2 GP I antibodies and raising the threshold of aCL to more than 40 units as positive (to avoid false positive results).^29^ For LAC, though any positivity is considered significant, a two-step test method using the diluted Russel viper venom time (dRVVT) as the primary phospholipid dependent test of coagulation has been recommended.^30^ It has been shown that use of dRVVT test for LAC is more sensitive for the detection of anti-β2 GP I antibodies.^31–33^

In a real-life scenario, it can be difficult and expensive to get all the aPL tests done in all patients. It is more difficult to confirm the presence of these aPL after 12 weeks: hence the heterogeneity and inconsistency of data on patients with APS. A practical approach will be to test for LAC and aCLIgG, and only if those are negative, to go for anti-β2 GP I antibodies. As LAC has highest association with thrombotic and pregnancy outcomes,^30,34^ detection of even weak levels of LAC should be considered significant. Anti-cardiolipin IgM is now considered of doubtful clinical significance. Testing for other antibodies like anti-prothrombin, anti-annexin V, anti-domain I antibodies are best suited for research laboratories. Ordering all three aPL tests can be useful in stratifying the aPL carriers for treatment decisions.

If a patient is already on anticoagulant, it is recommended that solid phase assays like aCL and anti -β2 GP I antibodies be done. However, some authors have reported that warfarin does not interfere with LAC detection tests done with dVVRT ratio method.^35^ Others have described methods to tests LAC in patients on anti-coagulation.^36^ Also, various studies have tried to stratify the risk of thrombosis (initial or recurrent) and pregnancy loss based on aPL profiles and risk scores like Global APS score or GAPSS.^37^ GAPSS is based on independent thrombosis and pregnancy loss risk factors and has proven in different cohorts of primary and secondary APS to be associated with higher risks of thrombosis and pregnancy loss.^38^

## CLINCAL FEATURES AND SUBGROUPS

The clinical features can be divided into criteria and non-criteria manifestations. The criteria manifestations are thrombotic and obstetric. Though thrombosis can involve almost any part of the vascular territory, it most commonly involves the venous system of lower limbs presenting as deep vein thrombosis, followed by pulmonary embolism. Central nervous system is the most common site of involvement in the arterial circulation presenting as transient ischemic attacks or ischemic stroke. The frequency of involvement of various parts of vascular system is best reported from the Europhospholipid cohort.^39^

The presence of thrombosis has to be documented either by established imaging method (Ultrasound Doppler, CT or MRI angiography) or histopathology (presence of thrombus in the absence of vessel wall inflammation).^26^

Obstetric APS is characterized by recurrent early pregnancy loss or single late pregnancy loss (>10 weeks gestation) or premature delivery (<34 weeks gestation) due to eclampsia or preeclampsia in the presence of aPL.^1,40^ These losses have to occur in the absence of maternal or paternal genetic abnormality in a morphologically normal fetus.^1^

Various non-criteria manifestations of APS have been described in literature.^40^ The frequencies of manifestations are listed (Table 2).

**Table 2:** Frequency of non-criteria manifestations of APS.

Hematological (thrombocytopenia/ hemolyticanemia): 64%
Cutaneous (lividoreticularis/ ulcers): 33%
Neurological (Chorea, decreasd cognition, myelitis): 27%
Cardiological (valvular thickening, NBTE): 8.2%
Renal (APS nephropathy): 6.4%

Thrombocytopenia has been described in initial case reports of the disease decades ago. Other manifestations occurring frequently are livedo reticularis, skin ulcers, nephropathy etc. These manifestations can occur with or without underlying criteria manifestations. The burden of these manifestations is highlighted by the fact that most of the patients having these features are on chronic steroid therapy.^41^ Moreover, none of the guidelines and very few studies have described the treatment approach for the non-criteria manifestations. With the recent understanding of the pathophysiology of these features, newer agents acting on complement pathways and B-cell pathways are being studied for efficacy and safety in treating non-criteria manifestations.

**Figure 2.**
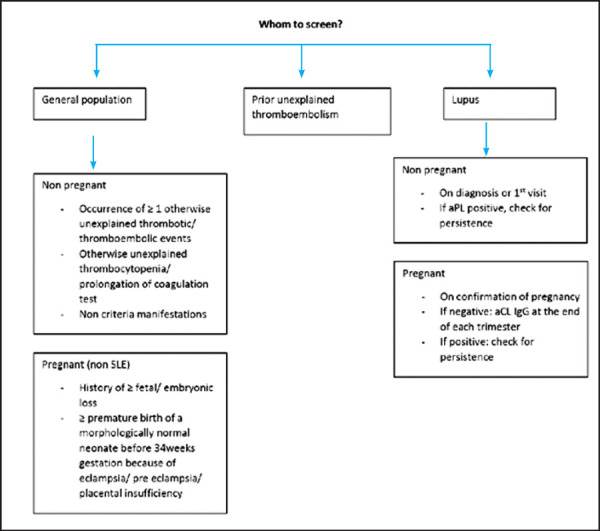
Screening for anti-phospholipid antibodies.

Among various non-criteria manifestations, APS nephropathy warrants special attention because of better understanding of its pathophysiology. APS nephropathy may occur in primary APS or in association with lupus. When it occurs in association with lupus, careful consideration should be given to differentiate the condition from lupus nephritis. APS nephropathy usually presents with accelerated hypertension and proteinuria; it rarely causes renal failure. Whenever possible a biopsy should be obtained to differentiate between the two conditions because of the different implications in the treatment plan.^41,42^ The use of screening tests for antiphospholipid antibodies in various clinical scenarios is summarized (Figure 2).

For the purposes of simplifying the treatment strategies, patients can be categorized into clinical subgroups. Each of the subgroups can have patients in two categories: primary APS (50%) or APS with underlying connective tissue diseases (usually lupus in another 35%). Those patients with only aPL antibodies but without any vascular event or pregnancy morbidity are classified as aPL carriers. Patients with evidence of vascular thrombosis and persistent aPL positivity are thombotic APS. This group can have three different presentations: venous APS, arterial APS, and recurrent venous APS. Similarly, patients with bad obstetric history as described in criteria with persistent aPL positivity but no history of past or current thrombotic manifestation are classified as obstetric APS. Whereas patients with bad obstetric history along with current thrombosis or history of thrombosis of arterial or venous territory and persistent aPL positivity are obstetric and thrombotic APS. Some may not have definite features of APS but one or more non-criteria manifestations of APS may be present with aPL positivity. Such group of patients are categorized as non-criteria APS. A serious life-threatening variant of APS is catastrophic APS (CAPS) characterized by progressive multiorgan failure due to thrombotic microangiopathy occurring rapidly over a period of few days.

The outcomes and treatment approaches are different according to the clinical subgroups. There is not much difference between primary and secondary cases of APS except that certain clinical features like thrombocytopenia, arthritis and systemic symptoms are more common in those with secondary APS. Hydroxychloroquine is universally added to all patients with underlying lupus.

## TREATMENT

Most of the treatment guidelines focus on management of thrombotic and/or obstetric manifestations of APS. Any episode of acute thrombosis is managed like thrombosis without APS with intravenous heparin infusion. In this review, long term treatment will be described according to the clinical subgroup of patients mentioned above.

aPL carriers: Treatment of aPL carriers depends on presence of risk factors. No treatment is recommended for those without any risk factors. If the patient has high risk aPL profile (based on GAPSS or triple positivity for LAC, aCL and anti-D2 GPI antibodies),^43^ or planning pregnancy,^44^ aspirin may be used. Fetal monitoring during pregnancy along with multidisciplinary care is adviced.^44^ Hydroxychloroquine (HCQ) is added to aspirin in aPL carriers with lupus.^45,46^ Thromboprophylaxis is recommended in high risk conditions like surgery, immobilization, post-partum period.

Thrombotic APS: An acute episode of thrombosis should be managed the same as any other cause of thrombosis. Initially anticoagulation is done with unfractionated heparin (UFH) or low molecular weight heparin (LMWH) followed by oral anticoagulation with a vitamin K antagonist. In case of venous thrombosis, moderate intensity warfarin therapy (INR 2 to 3) lifelong is recommended. However, high intensity warfarin therapy (INR 3 to 4) for life is recommended for arterial thrombosis and recurrent venous thrombosis. If the patient develops recurrent arterial thrombosis despite anticoagulation, platelet inhibitors (single or combination use of aspirin, clopidrogel, prasugel etc.) may be added to high intensity warfarin.^47^ Addition of hydroxychloroquine, or a statin to warfarin; use of a different anticoagulant, such as low-molecular-weight heparin; and a combination of these approaches has also been practiced by few.^48^

Obstetric APS: Risk of thrombosis and morbidity increases in pregnancy. Aspirin is initiated before planning pregnancy and continued throughout pregnancy till one week before delivery. LMWH or UFH in prophylactic dose (1 mg/kg once a day) subcutaneously is given at the confirmation of pregnancy. This has to be continued throughout pregnancy and stopped 12 hours before delivery. Heparin has to be restarted on first postpartum day for six weeks.^44^

Obstetric/Thrombotic APS: It is managed as obstetric APS. Warfarin may be started on first post-partum day to maintain INR at 2 to 3 (venous thrombosis) or 3 to 4 (arterial thrombosis). In cases of high risk of thrombosis, intravenous heparin can be started after stopping LMWH and stopped four hours before caesarean section.^44^ In refractory obstetric APS, treatments like IVIG at 0.4 g/ kg per day for five days each month during the next attempted pregnancy has been tried.^49^ Plasmapheresis with repeated exchanges of three or four treatments per week starting at the 14th week of pregnancy and continuing until caesarean delivery at 34 weeks has also been tried by few authors.^50^ Recent report has shown safety and efficacy of low dose steroids administered till 14 weeks pregnancy to improve outcomes in cases refractory to anticoagulation.^51^

Non-criteria APS: There are no standard treatment recommendations for non-criteria manifestations of APS. Recent report from the RITAPS study has shown some benefit with rituximab in thrombocytopenia associated with APS. Previous reports have shown benefit with intravenous immunoglobulin in severe cases. Patients with severe nephropathy may benefit with rituximab.^52^ Aggressive blood pressure control and high intensity warfarin have been shown to be effective in thrombotic involvement of renal microvasculature.^53^ Studies have shown benefit with Eculizumab in renal transplant recipients of APS nephropathy.^54^ Similar results of improved graft survival have been reported with Sirolimus in post-transplant patients. Heart valve abnormalities (vegetations and/or thickening) are the most common cardiac manifestations of APS.^55^ Presence of valve thickening is treated with low dose aspirin alone whereas full anticoagulation with heparin followed by long term warfarin is recommended for those with vegetations, thrombus or evidence of embolization. It is interesting to note that almost 20% patients with pulmonary hypertension due to chronic thromboembolism test positive for aPL. These patients might improve significantly with long term anticoagulation if identified.

CAPS: The best proven treatment for CAPS is combination of IVIG, plasmapheresis and intravenous heparin.^45,48^ In refractory cases, rituximab has shown its effectiveness.^48,56,57^ Studies are being carried out for effectiveness of eculizumab in this condition.

Alternative Anticoagulants: Non-coumarin group of anticoagulants have been studied in cases of deep vein thrombosis. The literature however is meagre in patients with APS. These agents include direct thrombin inhibitors like rivaroxaban, apixaban and dabegratan; and indirect inhibitors like fondaparinux. Recent RAPS study has shown that rivaroxaban is non-inferior to warfarin and safe in treatment of patients with APS.^58^ However, the TRAPS trial demonstrated increased risk of thromboembolic events and bleeding with rivaroxaban than warfarin.^59^ There are no systematic data yet for other drugs in this group.

These agents have minimal food and drug interactions and have predictable anticoagulation than warfarin making them beneficial in patients taking multiple medications and in those having difficulty in monitoring INR levels. However, as the anticoagulant effect cannot be monitored, they would not be the drug of choice for non-compliant patients. The anticoagulant effect of these drugs cannot be reversed easily and hence is avoided in patients requiring frequent discontinuation (eg. for procedures).

**Figure 3.**
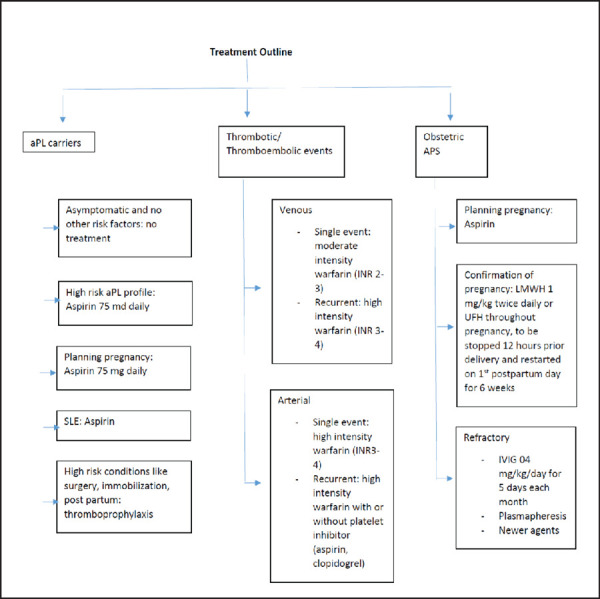
Treatment summary for thrombotic and obstetric APS.

A summary of treatment approach in various subtypes has been summarized (Figure 3).

Newer Agents: Many newer drugs such as Eculizumab, rituximab, sirolimus are being tried for the treatment of APS and its complications. Supportive drugs like statins and hydroxycholorquine also have some proven benefits.^60^ Eculizumab is a monoclonal antibody against C5a complement component which medicates the main pathogenic role in complement mediated injury. Few studies have shown efficacy of eculizumab in post-renal transplant recipient.^54,61^ Rituximab, an anti-CD 20 antibody, inhibits the Bcells and have shown to be effective in APS nephropathy, haemolytic anemia, thrombocytopenia and CAPS.^52,57,62^ A peptide molecule, TIFI binds to the domain V of D2 GPI, thus preventing its binding to the phospholipids on the endothelial cell surface. This inhibits the activation of complement as well as intracellular signaling pathways that activate the endothelial cells.^63^ Sirolimus, a calcineurin inhibitor, acts by inhibition of mTOR pathways that are responsible for expression of tissue factors. It has been found to increase the graft survival in post-renal transplant patients (74% vs 11%).^64^ The inhibitory effect of hydroxychloroquineon toll-like receptors helps in inhibiting the activation of monocytes and reduces the release of proinflammatory cytokines and tissue factors. This role has been tested and proven useful in model of primary APS patients who do not have lupus.^45,65,66^ Statins may also be used as an adjuvant therapy. Though not abundantly uses for APS, statins inhibit endothelial cell nitric oxide synthetase (eNOS) and helps in inhibiting activation of endothelial cells. It also has inhibitory action on the activation of monocytes.

## CONCLUSIONS

Anti-phospholipid antibody syndrome is a complex disease characterized by clinical features of thrombosis and pregnancy losses accompanied by the presence of antiphospholipid antibodies. With the identification of new antigens and their implications in triggering the clinical manifestations, newer treatment strategies and therapeutic agents are being developed. Most of the management guidelines focus on the prevention and treatment of thrombotic and obstetric manifestations of the syndrome and the anticoagulation paradigm has not changed over years. Immunosuppressants and newer biological agents have been reported to be successful in treatment of life-threatening and non-criteria manifestations. However, no definite guidelines can be given based on available literature. Addition of hydroxycholoquine with or without immunosuppressants to regular anticoagulation is usually done in cases of APS with underlying lupus.

## Conflict of Interest

**None.**
